# A Review: Developments in Hardware Systems of Active Ankle Orthoses

**DOI:** 10.3390/s24248153

**Published:** 2024-12-20

**Authors:** Praveen Nuwantha Gunaratne, Hiroki Tamura

**Affiliations:** 1 Interdisciplinary Graduate School of Agriculture and Engineering, University of Miyazaki, 1-1 Gakuen Kibanadai-nishi, Miyazaki 889-2192, Japan; 2 Faculty of Engineering, University of Miyazaki, 1-1 Gakuen Kibanadai-nishi, Miyazaki 889-2192, Japan

**Keywords:** active-ankle orthoses, rehabilitation, actuation modes, control strategies

## Abstract

Active ankle orthoses which have been designed over the past few years by diverse sources were critically reviewed in this paper. It begins by providing an overview of the anatomy of the ankle joint complex, establishing a basis for understanding the subsequent discussion on the research challenges and design difficulties associated with developing active ankle orthosis devices. The review systematically examined the mechanisms, actuation methods, and control strategies utilized in these orthosis devices. This covers various control strategies, including Electromyography (EMG)-based, adaptive, and modular control systems, emphasizing their importance in achieving precise and user-intended movements. By integrating insights from recent studies and technological innovations, this paper provides a holistic view of the progress in active ankle orthoses. The paper concludes with design recommendations aimed at overcoming existing limitations and promoting further development of advanced active ankle orthosis devices for future research.

## 1. Introduction

With the increase in the global elderly population and for the prevalence of neurological or muscular disorders, there is a growing interest in active orthoses within the medical industry to restore patient mobility and address healthcare issues related to locomotion difficulties [1]. There are three categories of robotic exoskeletons, which can be represented as upper-extremity, lower-extremity, and full-body systems. The primary usage of exoskeletons/active orthoses can be recognized as gait rehabilitation, power augmentation, and motion assistance. Active orthoses focusing on the lower extremity of the human body are designed to support the anatomical functions of lower extremity [2]. Normally, seven degrees of freedom (DOF) per limb is taken by the hip, knee, and ankle joints. With tri-planar motions, the ankle joint complex is more critical for the locomotion [3,4]. Recent advancements in active ankle orthoses have seen the incorporation of novel materials, actuators, and sensors. Modern ankle joint orthoses aim to balance functionality with wearer safety [5]. However, there is a need for further enhancement in their performance regarding metabolic energy savings, ergonomic conformity, and agility. A comprehensive understanding of these systems’ features and characteristics is essential to overcoming these limitations.

This comprehensive study focuses, from a research perspective, on active ankle orthoses developed through the mechanical and control systems. To provide a broad understanding, the review focuses on active ankle orthoses utilizing various state-of-the-art technologies, examining the latest designs to identify current paradigm shifts. Recent developments incorporate most electrical actuators with various transmission systems [6]. Belt drives, Bowden cable drives, and harmonic drives are notable among those transmission systems. Since the ability of storing passive energy in the spring element, and their low output impendence, Series Elastic Actuators (SEAs) are very important [7]. However, improvements in SEA technology are necessary to address performance drawbacks and sensor integration issues. Pneumatic actuators also show promise, offering advantages over electric actuators by closely mimicking biological muscles and reproducing joint stiffness properties. This makes them particularly suitable for applications requiring higher precision in joint movements [8]. While modern ankle orthoses primarily focus on actively supporting plantarflexion and dorsiflexion, there is potential for hybrid actuation methods to support multiple DOF, addressing key design challenges [9].

Ankle orthoses are occupied by a series of control techniques. There should be several requirements, but the most distinctive criteria are aligned with the intention of the user. Although the EMG-based control mechanism has greater potential, there will be some obstacles because of the variations on the EMG signal generation across users [10]. This has been particularly problematic for patients with muscular disorders. To address these challenges, integrating separate pressure or force sensor (force sensitive resistor)-based control strategies alongside EMG control can be beneficial. Adaptive controller architectures have proven successful in assisting user movements under varying conditions. For controlling multiple DOF, modular controllers are preferable to mitigate the complexity, as a centralized single controller may become overburdened [11].

The stability defined as a significant aspect in terms of control system design. Further, research has demonstrated that the result of the low robot/human interaction with high-frequency and high-amplitude external anxiety also plays a significant role in orthosis performance. Enhancing the functional performance of robotic devices can be achieved through improvements in both actuation methods and control systems. Hybrid active ankle orthoses have been recommended considering the nature of the articulation methods, modular control system, and the high and efficient power transmission in order to achieve the multiple DOF motions. This offers a high degree of agility in order to provide support to the wearer accommodating the specific limitations within the assistive technology [12,13,14,15]. 

Over the past few years, several active ankle orthoses have been developed primarily for rehabilitation purposes. As these devices envelop the human body to provide relevant power assistance, the kinematic design of the orthoses must be compliant with the ankle joint complex. In this case, the next section addresses the design difficulties arising from the perspective of the anatomical and biomechanical complexity of the ankle joint. The active ankle orthoses can be arranged broadly according to different applications with various actuation modes and control strategies, which will be discussed in later sections.

## 2. Design Considerations of Active Ankle Orthoses

### 2.1. Anatomy of Ankle Joint Complex

The plantarflexion/dorsiflexion, inversion/eversion, and abduction/adduction are the three most significant ankle joint movements. During the stance phase of walking, plantarflexion predominantly contributes to the energy required for propulsion. The range of motion for each DOF is as follows: plantarflexion (PF) spans 55 degrees to dorsiflexion (DF) at 20 degrees, inversion (IN) ranges from 23 degrees to eversion (EV) at 12 degrees, and abduction (AB) extends from 20 degrees to adduction (AD) at 25 degrees. It is noteworthy that several research papers indicate that range of motion (ROM) may exhibit variations influenced by factors such as age and gender [16,17].

The primary movements of the ankle joint occur at the talocrural (tibiotalar) and subtalar (talocalcaneal) joints. The talocrural joint facilitates plantarflexion/dorsiflexion. The subtalar joint permits inversion/eversion and abduction/adduction motions, supporting tri-planar movements [18]. The functional unit with the subtalar joint is the transverse tarsal joint and the subtalar joint contributes to foot inversion/eversion. The anatomical subtalar joint shares a common axis. An array of muscles works together on foot segment movements during locomotion. Furthermore, the understanding of the intricate interplay between the muscles and their tendons is crucial for comprehending ankle joint dynamics and designing effective active orthoses [19]. Moreover, ongoing research exploring the distinctions of muscle activation patterns and their implications for ankle joint function can further enhance the development of tailored orthoses to address various gait abnormalities and mobility impairments.

### 2.2. Design Difficulties of Active Ankle Orthoses

**Figure 1:**
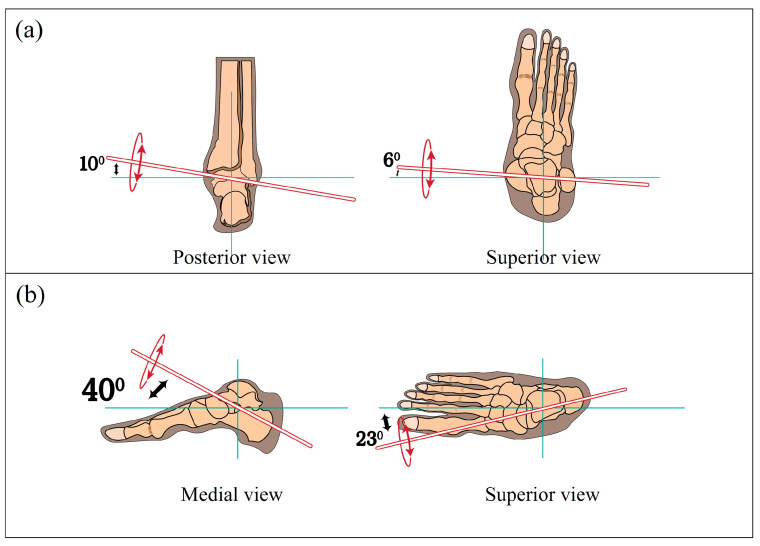
The oblique axes of rotation in relation to the anterior-posterior and medio-lateral body axes, as observed from different views: (**a**) oblique axis of plantarflexion/dorsiflexion movement (represents in Red); (**b**) oblique axis of inversion/eversion movement (represents in Red).

The anatomical complexity of the ankle joint creates a unique challenge in order to fit an orthosis comparatively with other lower limb joints. The oblique orientation of these joints’ anatomical axes, as illustrated in Figure 1, enables multidimensional foot movements, including pronation and supination during walking. 

Confining these motions to a single plane, mostly on the longitudinal/sagittal plane, can result in abnormal joint movements, inadequate muscle engagement, and increased energy loss [20]. During locomotion, the ankle and foot must provide both support and flexibility to adapt to changes in terrain and absorb shocks. The mechanical stiffness of these joints plays a crucial role in maintaining stability and facilitating efficient movement [21]. Mechanical stiffness at the ankle and foot is influenced by factors like ground irregularities, body weight, and walking speed. The overall capability of the orthosis as an adjusting system is an important factor in order to reassess the space to accommodate the biological joints stiffness when performing different activities. The joint instability or injury are the results of poor stiffness adjustments [22]. Furthermore, when it comes to everyday use, the portability of such developments should also be ensured. Achieving a balance between performance and portability remains a critical consideration in initial stages of such active orthosis designs.

### 2.3. Anthropometric Design in Active Ankle Joints

An anthropometric design focuses on the physical dimensions, proportions, and variability of the human body to create systems or devices that fit and align well with users’ anatomy. Having identical dimensions, joint ROM values, adaptability to user’s anatomy, ergonomic fit, and material and structural compatibility are some of the key criteria considered in anthropometric designs [23]. For an active ankle joint orthosis, this design approach is critical as it ensures the orthosis is appropriately scaled to the user’s body, providing a correct fit and optimal weight distribution at the lower extremities. Furthermore, it is essential for maintaining proper balance and minimizing pressure points to prevent any discomfort. In general, the use of rigid materials or designs that do not adapt well to individual biomechanics can lead to restricted motion or unnatural gait patterns and discomfort. Addressing these limitations through advanced anthropometric design can significantly improve usability, comfort, and user satisfaction, ensuring the orthosis aligns seamlessly with human anatomy [24,25].

## 3. Mechanisms and Actuation Methods

These devices incorporate various mechanisms and actuation methods to deliver precise and adaptive support. The primary mechanisms include hinge joints that mimic the natural movement of the ankle, sensor systems that monitor the user’s motion and interaction with the environment, and finally the control systems that process this data to adjust the device by mimicking the user behavior. Basically, the actuation methods typically involve motors and pneumatic or hydraulic actuators that provide the intended force (torque) to operate the ankle joint complex, allowing for dynamic assistance during different activities such as walking, standing, and navigating in different environments/uneven terrains. Together, these components work in sync to enhance the user’s functional capabilities. Here, a summary of the actuation methods, including their advantages and limitations, is provided in Table 1 at the end of the detailed review of primary actuation systems.

### 3.1. Electrical Actuator-Based Systems

**Figure 2:**
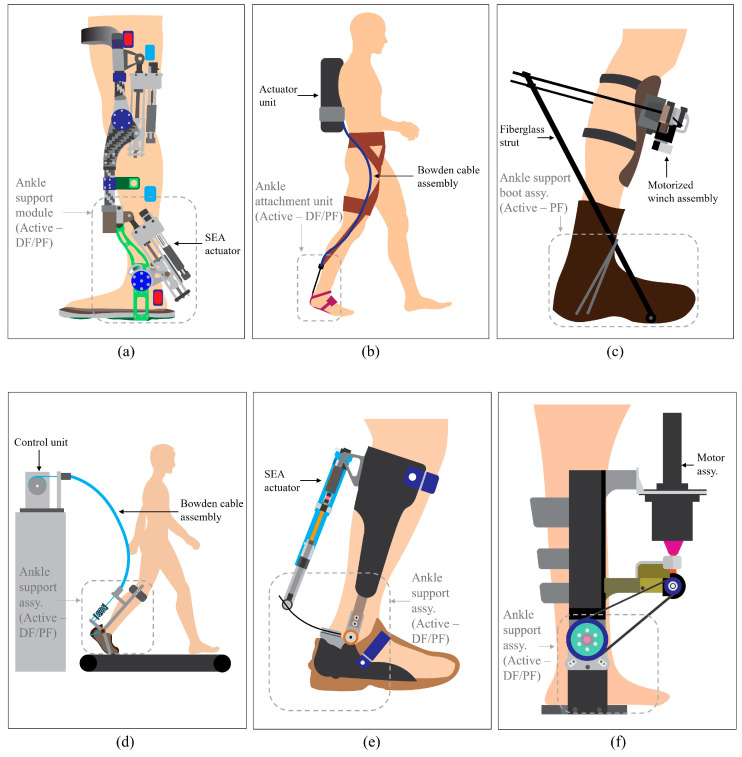
Conceptual designs of ankle orthoses utilizing electrical actuators: (**a**) knee-ankle-foot orthosis by National University of Singapore; (**b**) soft exosuit by Harvard University; (**c**) autonomous battery-powered exoskeleton by MIT; (**d**) an advanced orthosis by Carnegie Mellon University; (**e**) Achilles ankle exoskeleton by Delft University; (**f**) portable powered ankle-foot orthosis by Beijing Institute of Technology.

The portable knee-ankle-foot orthosis developed by the National University of Singapore (NUS) in 2013 was primarily designed for gait rehabilitation, targeting brain motor network reorganization of impaired individuals at outpatient and home settings. This orthosis is composed of ankle and knee modules separately as shown in Figure 2a [26], focusing primarily on sagittal plane movements. As it was developed using advanced composite materials, it has been able to achieve the modular, lightweight features. The notable area is the use of novel SEA, enabling precise torque control and safe interaction between orthosis and the patient. In this case, this design allows improved force control and energy storage. Here, the SEA is a type of electrically powered actuation method that incorporates elastic spring elements placed in series between the motor and load. The SEA actuation method has become prevalent in recent active ankle orthoses due to its ability to combine active and passive elements [27]. When compared to many other SEA-based designs, this knee-ankle-foot orthosis design enables the actuator to provide assistive torque effectively for operations with different torque requirements. In general, the SEA-based designs stand out for their enhanced energy efficiency, as the spring elements store and release energy effectively, reducing the overall energy consumption during locomotion. Here, the adaptability of SEAs to varying torque requirements could also make them particularly suitable for the dynamic and variable loads encountered in gait rehabilitation purposes. 

The Harvard soft exosuit developed in 2013, is a major improvement in active orthotic devices (see Figure 2b) [28]. As a flexible exoskeleton, this suit is made of soft, fabric-like material and uses a cable driven actuation system. This exosuit design targets improving mobility for patients with neurological disorders and assisting healthy people in carrying heavy items for military or outdoor tasks. Here, the Bowden cables connected to motors apply force to lower limb joints, providing up to 18 to 30% of the natural torque produced during normal gait. The suit applies forces to the ankle joint by selectively pulling on the cable at specific time intervals during the gait cycle. This pulling action generates a torque, which adds to the natural strength produced by the user’s muscles. The soft exosuit design ensures that the applied forces align with the natural motion of the ankle joint, preserving the user’s normal biomechanics. By pulling on the heel via the Bowden cable, the system generates a moment around the biological ankle joint. This action mimics the natural function of biological muscles by stretching pre-stressed tendons, which helps to absorb power passively and support muscle contractions actively. Compared to other electrical actuator systems, Bowden cable-based designs stand out for their lightweight and flexible structure, which reduces the bulkiness at lower extremities commonly associated with rigid actuator systems. This design allows greater adaptability to varying body sizes and shapes, making it suitable for a wider range of users. From an efficiency standpoint, the system’s ability to selectively engage during specific phases of the gait cycle ensures that energy is only used when needed, minimizing power consumption. Here, the soft exosuit’s design aims to enhance the wearer’s mobility by providing both passive and active assistance. The passive extension supports power absorption, while the active assistance aids muscle contractions, thereby reducing the metabolic cost of walking [29]. This dual approach allows for a more natural and efficient gait, which is particularly beneficial for individuals requiring gait rehabilitation or augmentation. In terms of cost, these kinds of systems generally offer a more economical alternative to fully motorized systems, as the cable-driven mechanism requires fewer complex components. Maintenance is also simplified due to the modular nature of the cable system, further contributing to cost-effectiveness. This combination of adaptability, efficiency, and affordability makes Bowden cable-based active orthoses a compelling choice for both clinical and commercial applications. By integrating these advantages into the design, the soft exosuit effectively addresses many of the limitations of conventional electrical actuator systems, ensuring a balance between performance and practicality.

The MIT autonomous battery-powered exoskeleton developed in 2014 aimed to reduce the metabolic cost of walking particularly during tasks involving heavy load (see Figure 2c) [30]. A motorized winch, mounted on the shin, pulls on fiberglass struts attached to the boots; these struts act as an extension of the user’s foot, creating a torque around the ankle joint during the push-off phase (plantarflexion operation) of normal gait. When the fiberglass struts are tensioned by the winch, they provide a rigid, supportive lever for the foot. This design maintains a lightweight profile and inertia by avoiding bulky actuator attachments directly to the foot. Furthermore, it emphasizes autonomous operation, which allows the active orthoses to function independently without external tethering, enhancing their practicality for real-world applications. Its lightweight architecture is crucial in minimizing the additional load on the user, thereby improving comfort and reducing fatigue during extended use. Ergonomic conformity ensures that the device aligns well with the user’s natural movements, thereby enhancing both the effectiveness of assistance and the wearer’s overall experience. Key innovations in this exoskeleton include the use of advanced materials and the integration of state-of-the-art control systems, which collectively contribute to its successful implementation.

The Carnegie Mellon University ankle orthosis developed in 2014 (see Figure 2d) serves as a high-performance research tool designed to assist with human walking [31]. The primary goal of this orthosis is to assist the wearer’s ankle during walking by mimicking natural ankle movements and augmenting the forces required for gait. It is also designed as a testbed for examining robotic assistance techniques. Unlike some other orthoses that use direct mechanical actuators or rigid frameworks this system uses a flexible Bowden cable tether to transmit power from the off-board motor to the exoskeleton end-effectors. Use of the Bowden cable reduces the weight of the wearable parts assembly, making it more comfortable for the user while still allowing for effective force transmission [32]. Furthermore, the design of this orthosis emphasizes research versatility as its modularity, precise torque control, and ability to simulate human ankle dynamics allow it to serve as an ideal platform for testing various robotic assistance strategies and developing rehabilitation interventions.

The Achilles ankle exoskeleton developed by Delft University in 2015 (see Figure 2e) [33] assists with the push-off phase of normal gait by mimicking the function of the Achilles tendon. The system incorporates a spring mechanism as a part of its actuation method. Which serves a similar purpose to a SEA by storing and releasing elastic energy to mimic the behavior of the human Achilles tendon during walking. The lever mechanism stores energy during the early stance phase of the gait cycle similarly to how the Achilles tendon stores elastic energy when stretched under load. During the push-off phase, the spring releases stored energy combined with the power from the motor in order to give the required assistance, which results in reducing the load on the user’s muscles. This hybrid approach of using a SEA led to a combination of precise torque control and better energy storage/release dynamics. 

A portable powered ankle-foot orthosis developed by the Beijing Institute of Technology in 2015 (see Figure 2f) [34] was designed to assist on normal gait by supporting the sagittal plane ankle joint mechanics (dorsiflexion and plantarflexion). This design employs an electric motor assembly as its primary actuator, effectively providing the necessary energy for predicted ankle movements. The power transmission of this orthosis includes a sophisticated transmission system combining harmonic drive, bevel gear, and synchronous belt units. These components work closely together to deliver power to the required ends. The electrical motor-based designs demonstrate significant advantages in terms of precision and torque output, making them particularly effective for assisting with controlled, dynamic movements that are required in gait rehabilitation exercises. Furthermore, these systems show higher adaptability for varied user needs and gait patterns, allowing integration with sophisticated control systems for real-time monitoring and adjustments. Despite its ability to deliver high torque, the bulkiness and weight of the system significantly limit its compatibility for everyday use. Basically, this highlights a common challenge in the design of active orthosis devices which is the balance between performance and the necessity for portability or user comfort. 

### 3.2. Pneumatic Actuator-Based Systems

**Figure 3:**
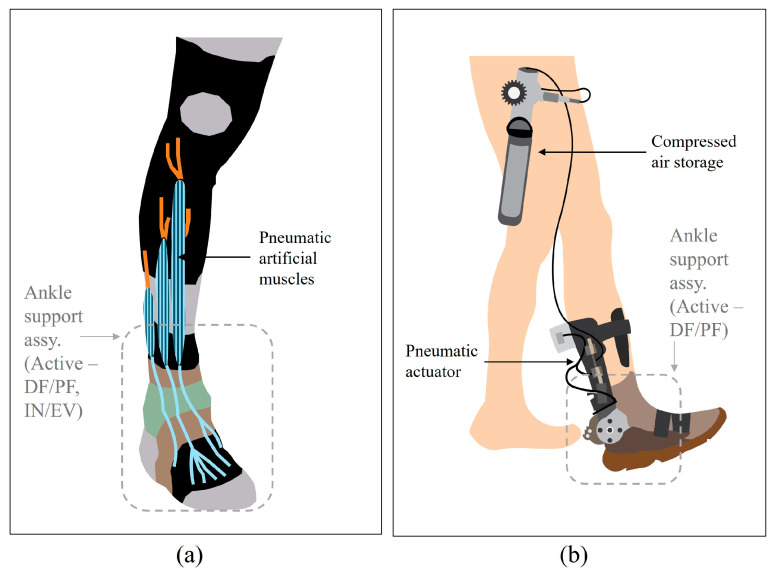
Conceptual designs of ankle joint orthoses utilizing pneumatic actuators: (**a**) bio-inspired active soft orthosis by Harvard University; (**b**) portable powered ankle-foot orthosis by University of Illinois.

A bio-inspired soft orthosis developed by Harvard University in 2011 (see Figure 3a) [35] is a soft robotic device designed for ankle/foot rehabilitation purposes. The pneumatic artificial muscles (PAMs) work as their primary actuation mechanism. Such artificial muscles mimic the function of biological muscles, allowing accurate control over plantarflexion/dorsiflexion and inversion/eversion movements at the ankle joint complex. PAM-based systems also excel in adaptability, as they can be easily scaled and adjusted to meet different user requirements. Their compliance and ability to absorb shock loads make them particularly suitable for patients undergoing rehabilitation, where comfort and safety are critical. When compared to traditional electrical actuator-based systems, PAM-based designs offer distinct advantages in terms of energy efficiency and natural movement replication. By leveraging compressed air to generate force, PAMs provide smooth and continuous motion that closely mimics the behavior of human muscles. This capability to produce adaptive and variable forces enhances the overall performance of this active ankle orthosis, particularly in applications requiring precise control over complex joint movements in sagittal and frontal planes. Despite its remarkable performance, notable limitations of this kind of orthosis device are the bulkiness and initial cost, when the pneumatic circuit is considered. These constraints make boundaries for its potential for widespread clinical applications. 

The University of Illinois developed a portable powered ankle-foot orthosis (PPAFO) in 2012, depicted in Figure 3b [36]. This device was designed to assist walking by providing power to the ankle joint operations using compressed carbon dioxide (CO_2_). The system includes several key components such as a dual-vane pneumatic actuator for sagittal plane ankle movements (dorsiflexion/plantarflexion), and a compressed CO_2_ regulatory system with a gas storage safely mounted on the user’s waist area. Despite its single plane operations, this system lies in its standalone power system, which makes it more portable for the use of long run. Furthermore, its energy-saving design incorporates a mechanism to recycle pneumatic energy, improving efficiency and extending the runtime. In this case, the reliance on compressed gas as a power source eliminates the need for heavy batteries or motors. Furthermore, this system demonstrates remarkable efficiency by providing high power-to-weight ratios by making the system lightweight and compact for outdoor or long-term rehabilitation scenarios. The adaptability is increased by the system’s modularity and compact design allowing it to accommodate a wide range of users with varying anatomical and functional requirements. However, a few challenges remain, such as ensuring consistent gas supply and pressure for outdoor use. 

### 3.3. Hydraulic Actuator-Based Systems

The Berkeley lower extremity exoskeleton (BLEEX), developed by the University of California in 2005 [37], can be taken as a notable example for active ankle orthosis incorporating linear hydraulic actuators for mobility assistance. The BLEEX device features an ankle joint capable of all three distinct directional movements. While it does not fully replicate the anthropomorphic characteristics of ankle joint complex in its design, it has been able to match them with primary operations based on the design objectives while compliant on secondary ankle joint operations. The dorsiflexion/plantarflexion operation is facilitated by a specialized hydraulic mechanism equipped with linear hydraulic actuator for force control. Despite the size of the hydraulic actuator arrangement, these components exhibit remarkable strength, although their integration into wearable devices poses significant challenges. The adaptability of its hydraulic components allowing adjustable force output is vital for facilitating comfortable and safe locomotion while accommodating the additional demands of carrying payloads. Regardless of some of the inherent challenges in design, the overall system serves as a compelling demonstration of the potential benefits of hydraulic-powered ankle orthosis in improving mobility outcomes.

**Table 1:** Summary on advantages and limitations of different actuation methods of active ankle orthoses.

Actuation Method	Example Orthoses Device	Advantages	Limitations
SEA [33,38]	Portable knee-ankle-foot orthosis by NUS [26]	Less energy consumption, delivery of variable torque, and modular design	Bulkiness at the lower extremity
Bowden cable-driven [31]	Soft exosuit by Harvard University [28]	Lightweight and flexibility	Complexity of the overall system
Motor with cable/gear driven [34,39,40,41]	Autonomous battery- powered exoskeleton by MIT [30]	Precise torque output, higher adaptability	Bulkiness and weight
PAM [42,43]	Bio-inspired soft orthosis developed by Harvard University [35]	High energy efficiency, natural-like movement	Bulkiness and initial cost
Pneumatic actuator	Portable powered ankle-foot orthosis by University of Illinois [36]	High energy efficiency, lightweight, and compact	Challenging to ensure stable gas supply and pressure
Hydraulic actuator	Berkeley lower extremity exoskeleton by University of California [37]	Adjustable torque output	Bulkiness and complexity of the overall system

## 4. Control Methods

Different objective-based active ankle orthoses utilize a variety of control strategies to provide effective and adaptive assistance to individuals with mobility impairments. These include Proportional-Derivative (PD) control for maintaining desired ankle angles, impedance control to adjust resistance based on movement, and Model Predictive Control (MPC) for anticipatory adjustments. Adaptive control continuously fine-tunes the parameters in response to the user and user/environment, while event-driven control synchronizes assistance with the gait cycle. The EMG-based control leverages muscle activation signals for intuitive responsiveness. In many cases, the force/pressure control adjusts the support in real-time based on exerted force and the neural network or machine learning-based control strategies have also been used to personalize assistance through user data analysis over time. Furthermore, the hybrid control strategies combine some of these general methods to enhance the robustness and flexibility, when facilitating varied objectives and motion phases [44,45].

### 4.1. EMG-Driven Control Strategy

The control methods for these ankle orthoses are usually based on the precise prediction of the user’s intended motion in real time. As the skin surface EMG directly reflects different activity levels of the user’s muscles, it has been considered as one of the primary data sources for many developments. In the case of flexible motion assistance, the ankle joint motions must be precisely estimated based on EMG signals from the lower limb muscles [46,47]. The SEA-based portable knee-ankle-foot orthosis developed by the National University of Singapore in 2013, employs an EMG-based control with PD [26]. Here, the control is achieved via a feedback loop that integrates data from multiple sensors to monitor and adjust movements during each gait cycle. The EMG sensors track the activation of muscles for ankle joint movements and the joint angle sensors; foot pressure sensors and Inertial Measurement Units (IMU) have been employed to gather data on different phases within the gait cycle. As a whole, the system uses data from all of these sensors to adjust the level of assistance dynamically, in real-time. Through this integrated sensor feedback and adjustment systems, the rehabilitation process may become more personalized and responsive to the individual condition of the patient while having a continuous improvement in the effectiveness of the treatment. One of the primary challenges is the computational complexity associated with processing and analyzing real-time EMG signals. As EMG signals can be noisy and prone to interference from non-target muscles or many other environmental factors, it demands sophisticated filtering and signal processing algorithms. These computational challenges can lead to delays or errors in predictions of the user’s intended motion, eventually resulting in discomfort for the user. Another limitation is the dependence on user training. EMG signals are highly individual, with significant variability across different users in terms of their muscle activation patterns. As a result, the system often requires the user to undergo training sessions to fine tune the device’s responses to their specific muscle signals which are more time consuming. Despite these challenges, advances in machine learning and adaptive control algorithms hold the potential to address these limitations, making EMG-driven control strategies more reliable and efficient.

### 4.2. Event-Driven Control Strategy

The event-driven control strategy works by responding to a specific gait-related event or phase while ensuring a precise and efficient assistance during movement. In this case, the system continuously monitors sensor inputs to identify a specific event in the user’s gait cycle. IMUs, foot pressure sensors, and joint angle sensors are most commonly used for such event detection. Once an event is being captured by the system, the device is capable of providing the targeted assistance. The control algorithm ensures that the assistance is delivered at the appropriate moment and magnitude. This control strategy can be easily adapted with the user’s specific gait characteristic while accommodating for some dynamic changes [48]. As the assistance is provided only during relevant events/gait phases, the overall power requirement is at a minimal level. The BLEEX device developed by the University of California [37] uses an event-driven control strategy to adjust the level of assistance during walking. As per its design objectives, the system is set viable for supporting load during the stance phase or aiding foot lift in the swing phase. Overall, this approach makes the orthoses devices more responsive, user-friendly, and efficient for certain rehabilitation and load-carrying applications.

### 4.3. Model-Driven Control Strategy

The model-driven control strategy relies on a mathematical or computational model of a system to predict its behavior and assist with different human-like movements. Under the system modeling, a mathematical or dynamic model of the exoskeleton system is created based on joint kinematics, muscle/skeletal interactions and also considering some of the external forces acting on the body and device [49]. Based on the model, the control algorithms are developed to achieve the synchronized natural movements while minimizing the metabolic cost. Even these systems are primarily model driven, often integrated with sensor feedback in order to make immediate corrections on the deviations from the predicted movements. The Realistic Model Reference Computed Torque Control Strategy by Miami University [50] has successfully implemented a model-driven computed torque control strategy to manage joint dynamics accurately for neuro-rehabilitation applications. This approach has involved using a novel realistic model reference computed torque controller to predict torque values based on the gait reference trajectories, while overcoming some of the limitations present in inverse dynamics models. Furthermore, it shows the adaptability and precision of model-driven control strategies in orthoses devices, offering better performance in tracking and handling uncertainties compared to other control strategies. 

### 4.4. Impedance-Driven Control Strategy

The impedance-driven control strategy regulates the interaction between the user and the device to enable natural and safe movements. This approach models the interaction between human and the device as a mass-spring-damper system, where parameters like stiffness and damping are dynamically adjusted based on the feedback from sensors monitoring certain muscle activities, force, and joint angles [51]. An active ankle foot orthosis (AAFO) by J. A. Blaya et al., 2004, [52] utilizing an impedance-driven control strategy that adjusts joint stiffness and damping dynamically during the gait cycle to improve walking mechanics for individuals with drop-foot gait. During the stance phase, the device minimizes forefoot collisions by mimicking natural torsional spring stiffness and later the joint impedance is reduced to allow powered plantarflexion for a natural push-off. During the swing phase, a spring-damper mechanism ensures a safe toe clearance.

### 4.5. Hybrid Control Strategy

This combines different control methods to improve the adaptability, efficiency, and responsiveness of the device in assisting or enhancing the user’s gait. In active-ankle orthoses, hybrid control strategies generally integrate feedback and adaptive control elements to help achieve smooth and natural movement. Galle, S. et al., 2017 [12] examined an ankle exoskeleton that combines adaptive control with impedance control to reduce the metabolic cost of walking. The adaptive control system adjusts the exoskeleton’s parameters in real time according to the user’s changing gait patterns, such as speed and stride length. In parallel, impedance control provides compliant support, ensuring minimal resistance and smooth movement, allowing the device to adapt seamlessly to different activity intensities, like transitioning from walking to jogging. This hybrid approach optimizes actuation timing and power based on user behavior and the gait phase, enhancing overall walking efficiency [53]. While the hybrid control strategy offers several advantages in terms of adaptability and efficiency, it also presents several limitations. A primary challenge is the computational complexity involved in real-time processing of multiple control inputs and adjustments. The system needs to simultaneously process data from various sensor inputs and adjust parameters according to the user’s intended motion while ensuring the actuation is synchronized with the different phases of movement.

## 5. Discussion

Active ankle orthoses are pivotal in rehabilitating ankle joint functions, employing sophisticated mechanisms, power systems, and control strategies. This paper delves into a comprehensive exploration of advanced active ankle orthoses, focusing on their technicality and emerging trends in the field. Dynamic use, user comfort, durability, and cost-effectiveness are some of the critical factors that define the intended use of these active ankle joint orthoses beyond their technical specifications. While technical elements such as the use of advanced materials, actuation methods, and control systems are essential for ensuring functionality, in general these intended use factors directly impact the orthosis’s practical application and overall user experience. The ability of the device to support dynamic movement, provide comfort during extended use, withstand sever conditions during daily activity, and remain cost-effective for a wide range of users is crucial for its success in real-world settings. Having a good balance between these user-centered factors with the necessary technical capabilities is essential for an active orthosis that is both effective and accessible.

The importance of anthropometric design in active ankle joint orthosis cannot be overstated, particularly when considering dynamic use and user comfort. Proper anthropometric design ensures that the orthosis fits the user’s unique body dimensions and biomechanical needs, which is critical for achieving optimal performance. The anatomical variability among different individuals necessitates personalized design or adjustability in designs to minimize any discomfort due to pressure points and ensure that the orthosis moves harmoniously with the user’s natural gait pattern. A well-tailored active ankle orthosis system also contributes to the device’s durability by preventing undue stress on specific parts of the joint. Furthermore, a correctly scaled orthosis facilitates better weight distribution, enhancing both stability and mobility during dynamic activities. When these factors are carefully addressed, the design can significantly reduce the risk of any skin irritations, joint misalignment, and muscle strain over time, which are common issues in ill-fitting equipment. However, most of the above discussed active ankle joint orthoses developments often overlook the importance of anthropometric design criteria. The ankle joint, being a complex structure with limited space compared to other lower extremity joints, presents unique challenges in achieving a proper fit. As a result, many devices fail to fully accommodate the anatomical complexity of the ankle joint, leading to compromised comfort and functionality. This issue needs to be addressed properly through future research by incorporating advanced manufacturing techniques, more precise control methods, and design architecture.

In hardware development, material selection plays a pivotal role in the functionality, durability, and comfort of active ankle joint orthoses. An ideal material should balance flexibility and rigidity, providing both the necessary support to maintain joint alignment and the flexibility required for dynamic motion. Materials such as lightweight composites and advanced alloys offer durability while maintaining a low profile, which is essential for ensuring the orthosis is not bulky for the user during motion. The biocompatibility and wear resistance of materials also need to be considered typically after the initial prototype phases, as these factors directly impact the device’s longevity and the user comfort. Striking the right balance between material properties and affordability/cost-effectiveness is crucial for ensuring that the orthosis remains both practical and economically viable.

The selection of appropriate actuation methods is another critical factor in determining the overall performance of an active ankle joint orthosis, especially when considering its dynamic use. In general, these actuators must be capable of producing smooth, precise movements that mimic the natural ankle joint movements. As discussed previously, various actuation methods, such as electric, pneumatic, and hydraulic systems, offer significant advantages in terms of responsiveness, control, and weight. Further, the energy consumption of such actuation systems must also be considered when moving into the next phase of the development cycle, to ensure that the orthosis remains functional over the intended period of time. Electrical actuators stand out as the predominant actuation method in many active ankle orthoses, leveraging diverse transmission systems. These actuators offer precise control over complex ankle movements, facilitating targeted assistance tailored to the wearer’s needs. The SEAs have also gained attention despite their inherent limitations, owing to their ability to store passive energy and maintain low output impedance. However, further advancements are important to optimize SEA performance and seamlessly integrate them into orthoses systems. In contrast, pneumatic actuators present distinct advantages, closely mimicking biological muscle behavior and offering inherent compliance essential for natural joint movements. Their ability to reproduce lower limb stiffness properties enhances user comfort and overall orthotic performance. Hybrid actuation methods are also emerging as a promising avenue for enhancing ankle orthosis functionality, enabling support for multiple DOF. This approach addresses critical design challenges, including ergonomic conformity, metabolic energy savings, and agility, thus revolutionizing active orthoses design paradigms. Therefore, the selection of the most appropriate actuation method is a balance between responsiveness, energy efficiency, weight, and cost considerations.

Development of effective control strategies is also essential for maximizing orthoses performance and user satisfaction. Advanced sensors and control algorithms play a key role in transforming the user’s motion intentions into precise actuation responses, ensuring that the orthosis adapts dynamically to different daily activities. The control system must be highly responsive to real-time feedback from sensors, which detect parameters such as joint angle, force, and pressure. In this case, a proper control system can significantly improve user comfort by minimizing the mechanical resistance during the swing phase or providing an additional support during the stance phase of the normal gait pattern. Based on many cases, it has proven that successful integration of EMG-based control strategy allows for natural human-like interaction, aligning orthotic actions with user intent. However, challenges such as EMG signal variability across different users and necessitate robust sensor integration and algorithm refinement. Combining pressure or force sensor-based control strategies with EMG control enhances orthotic responsiveness and adaptability to varying user needs and environmental conditions. Adaptive controller architecture further augments orthotic functionality by dynamically adjusting assistance levels to optimize user comfort and performance. The modular control architectures of many devices offer scalability and flexibility into the system, enabling independent control of multiple DOFs to enhance overall system robustness and usability. By leveraging these advanced control methodologies, it is clear that active ankle orthoses can effectively address the diverse needs of users and in the long run, they could significantly improve their quality of life. However, sophisticated control systems can increase both the complexity and cost of the active ankle joint orthosis. Ensuring that the control methods are not only effective but also cost-efficient is critical for making the orthosis accessible to a broader range of users. Additionally, user-friendliness is a key consideration as the control systems must be intuitive, requiring minimal input from the user while still providing adaptive, real-time feedback. Balancing advanced control features with affordability and ease of use is essential for improving the overall experience and practicality of the orthosis.

## Data Availability

As this is a review paper, no new data were generated or analyzed. All data referenced in this review are publicly available through the sources cited in the manuscript.
